# O Teste do Degrau de Seis Minutos como Preditor de Capacidade Funcional de Acordo com o Consumo de Oxigênio de Pico em Pacientes Cardíacos

**DOI:** 10.36660/abc.20190624

**Published:** 2021-05-06

**Authors:** Luiz Eduardo Fonteles Ritt, Eduardo Sahade Darzé, Gustavo Freitas Feitosa, Jessica Santana Porto, Gabriela Bastos, Renata Braga Linhares de Albuquerque, Cristiane Miura Feitosa, Thaissa Costa Claro, Eloisa Ferreira Prado, Queila Borges de Oliveira, Ricardo Stein

**Affiliations:** 1 Hospital Cárdio Pulmonar SalvadorBA Brasil Hospital Cárdio Pulmonar, Salvador, BA - Brasil; 2 Escola Bahiana de Medicina e Saúde Pública SalvadorBA Brasil Escola Bahiana de Medicina e Saúde Pública, Salvador, BA - Brasil; 3 Hospital de Clínicas de Porto Alegre Porto AlegreRS Brasil Hospital de Clínicas de Porto Alegre, Porto Alegre, RS - Brasil; 4 Universidade Federal do Rio Grande do Sul Porto AlegreRS Brasil Universidade Federal do Rio Grande do Sul, Porto Alegre, RS - Brasil

**Keywords:** Insuficiência Cardíaca, Consumo de Oxigênio, Capacidade Respiratória, Volume de Ventilação Pulmonar, Teste de Esforço

## Abstract

**Fundamento:**

O teste do degrau de seis minutos (TD6) é uma forma simples de avaliar a capacidade funcional, embora tenha sido pouco estudado em pacientes com doença arterial coronariana (DAC) ou insuficiência cardíaca (IC).

**Objetivo:**

Analisar a associação entre o TD6 e o consumo de oxigênio de pico (VO_2pico_) e desenvolver uma equação que estime o VO_2pico_ com base no TD6, bem como determinar um ponto de corte para o TD6 que preveja um VO_2pico_ ≥ 20 mL.kg^-1^.min^-1^

**Métodos:**

Nos 171 pacientes submetidos ao TD6 e a um teste de exercício cardiopulmonar, análises da curva ROC, de regressão e de correlação foram usadas, e um p < 0,05 foi admitido como significativo.

**Resultados:**

A idade média foi 60±14 anos, e 74% eram do sexo masculino. A média da fração de ejeção ventricular esquerda foi 57±16%; 74% apresentavam DAC, e 28%, IC. A média do VO_2pico_ foi 19±6 mL.kg^-1^.min^-1^, e o desempenho médio do TD6 foi 87±45 passos. A associação entre o TD6 e o VO_2pico_ foi r 0,69 (p < 0,001). Os modelos VO_2pico_ = 19,6 + (0,075 x TD6) – (0,10 x idade) para homens e VO_2pico_ = 19,6 + (0,075 x TD6) – (0,10 x idade) – 2 para mulheres poderiam prever o VO_2pico_ com base nos resultados do TD6 (R ajustado 0,72; R^2^ ajustado 0,53). O ponto de corte mais acurado para que o TD6 preveja um VO_2pico_ ≥ 20 mL.kg^-1^.min^-1^ foi de > 105 passos [área sob a curva 0,85; intervalo de confiança de 95% 0,79 - 0,90; p < 0,001].

**Conclusão:**

Uma equação que preveja o VO_2pico_ com base nos resultados do TD6 foi derivada, e foi encontrada uma associação significativa entre o TD6 e o VO_2pico_. O ponto de corte do TD6, que prevê um VO_2pico_ ≥ 20 mL.kg^-1^.min^-1^, foi > 105 passos. (Arq Bras Cardiol. 2021; 116(5):889-895)

## Introdução

Na doença cardiovascular, a capacidade funcional está diretamente relacionada ao prognóstico.^1^ O desempenho funcional, conforme determinado pelo consumo de oxigênio de pico (VO_2pico_) e medido por um teste de exercício cardiopulmonar (TECP), é o padrão-ouro e é utilizado para determinar o prognóstico de insuficiência cardíaca (IC) e seleção de transplante cardíaco, bem como para avaliar a resposta terapêutica.^2^^–^^4^ Pacientes com VO_2pico_ abaixo de 15 mL.kg^-1^.min^-1^ apresentam um perfil prognóstico pior, e aqueles com VO_2pico_ acima de 20 mL.kg^-1^.min^-1^ apresentam um perfil prognóstico melhor, independentemente da etiologia da IC e da função ventricular.^5^^,^^6^ Embora amplamente utilizado e validado, o TECP não está disponível na maioria dos centros, pois o equipamento é caro e é necessário que um médico especializado administre o teste e interprete seus resultados.

Uma alternativa ao TECP é o teste de caminhada de seis minutos (TC6), o qual é bem validado e apresenta boa correlação com o TECP em pacientes com cardiomiopatia.^7^ No entanto, o TC6 requer um longo corredor (com pelo menos 30 metros), o que pode limitar seu uso na prática comum.

O teste do degrau de seis minutos (TD6) é um teste simples no qual o paciente sobe e desce uma escada de 2 degraus por 6 minutos em cadência livre, e o número de passos é contabilizado. Não requer equipamentos sofisticados nem espaços grandes. Embora estudado em pacientes com doença pulmonar crônica e em indivíduos normai,8-11 não há dados sobre o desempenho do TC6 em pacientes cardíacos.

Os objetivos deste estudo foram: (1) analisar a associação entre o TD6 e o VO_2pico_, (2) desenvolver uma equação para estimar o VO_2pico_ com base nos resultados do TD6 e (3) determinar um ponto de corte para a categoria de baixo risco no TD6 (VO_2pico_ ≥ 20 mL.kg^-1^.min^-1^).

## Métodos

Neste estudo transversal, avaliamos pacientes encaminhados para reabilitação cardíaca entre maio de 2014 e setembro de 2017 que, conforme o protocolo clínico, foram submetidos a TECP limitado a sintomas e TD6 como avaliação basal no programa de reabilitação cardíaca do Hospital Cárdio Pulmonar, em Salvador, Brasil.

Os critérios de inclusão foram pacientes maiores de 18 anos com diagnóstico de doença arterial coronariana (DAC) ou IC, caracterizadas por infarto agudo do miocárdio prévio, angioplastia coronariana/implante de stent pós-cirurgia cardíaca ou vascular ou pacientes com dispositivos implantáveis, como marca-passos ou desfibriladores cardíacos. Esses indivíduos foram encaminhados ao programa de reabilitação cardíaca e submetidos a avaliação inicial com cardiologista e fisioterapeuta. O diagnóstico de DAC e/ou IC foi estabelecido pelo histórico médico (infarto agudo do miocárdio, DAC estável, revascularização do miocárdio ou angioplastia ou sintomas de dispneia ou angina), anormalidades eletrocardiográficas (ondas Q patológicas) e anormalidades ecocardiográficas (disfunção ventricular e anormalidades segmentares).

O critério de exclusão foi incapacidade de realizar o TECP ou o TD6. Pacientes com sintomas de angina ou isquemia em estágio inferior ao limiar anaeróbio também foram excluídos por não terem sido submetidos ao TD6.

Os dados clínicos e demográficos foram obtidos da avaliação cardiológica inicial no dia do TECP, incluindo o ecocardiograma mais recente (nos últimos 3 meses). O TECP e o TD6 foram aplicados separadamente, com 2 a 7 dias de intervalo.

O TD6 foi realizado em um degrau de 20 cm de altura coberto com borracha antiderrapante. Os pacientes foram instruídos a subir e descer o degrau o mais rápido possível por 6 minutos, sem usar os braços para se apoiar; pausas para descanso eram permitidas durante os 6 minutos.

O TECP limitado a sintomas foi realizado em uma esteira com um analisador de gases (Cortex, Leipzig, Alemanha) com medidas a cada respiração. Foi utilizado um protocolo de rampa individualizado baseado na classe funcional de cada paciente, com duração da fase de exercício direcionado entre 8 e 12 minutos. Os dados ventilatórios coletados foram tabulados e analisados em intervalos de 10 segundos.

Aspectos éticos: o protocolo do estudo foi aprovado pelo Comitê de Ética em Pesquisa Celso Figueiroa do Hospital Santa Izabel (processo 1.711.505). O estudo foi conduzido de acordo com a legislação nacional e internacional de pesquisa em humanos, incluindo a Declaração de Helsinque e a resolução 466/12 do Conselho Nacional de Saúde do Brasil. O consentimento informado foi dispensado, uma vez que o estudo utilizou apenas dados de prontuários médicos.

### Análise Estatística

Todas as análises foram realizadas no *software* SPSS, versão 25.0. As variáveis contínuas foram apresentadas como média ± desvio padrão para a distribuição paramétrica. O teste de Shapiro-Wilk e a inspeção visual dos histogramas foram utilizados para determinar a normalidade. As variáveis categóricas foram apresentadas em número ou porcentagem. A correlação de Pearson foi aplicada para determinar associações entre variáveis contínuas, e os gráficos de Bland-Altman foram utilizados para analisar sua concordância. Análises de regressão linear univariada e multivariada (após análise das suposições adequadas) foram realizadas para determinar a previsão de VO_2pico_ do modelo com base no TD6, o qual foi controlado para idade, fração de ejeção, sexo, presença de DAC ou IC e peso. A análise da curva ROC foi aplicada para determinar os melhores pontos de corte para prever VO_2pico_ ≥ 20 mL.kg^-1^.min^-1^. Um valor de p de < 0,05 foi considerado estatisticamente significativo.

## Resultados

A amostra total consistiu em 171 indivíduos. Suas características clínicas e demográficas são apresentadas na Tabela 1. A maioria dos pacientes apresentou classe funcional I ou II da New York Heart Association (NYHA) com VO_2pico_ médio de 19±6 mL.kg^-1^.min^-1^.

**Tabela 1:** Características clínicas e demográficas gerais da população

Variável	Resultado
Sexo masculino % (n)	74% (121)
Idade (anos)	60±14
DAC % (n)	74% (121)
Insuficiência cardíaca % (n)	28% (47)
Valvulopatias % (n)	13% (22)
Diabetes % (n)	25% (44)
Hipertensão % (n)	62% (102)
NYHA I, II, III%	53%/24%/10%
Inibidor de ECA-BRA % (n)	65% (110)
Betabloqueador % (n)	77% (130)
Estatinas % (n)	75% (128)
Fração de ejeção (%)	57±16
VO_2pico_ (mL.kg^-1^.min-1)	19±6
VO_2_ no limiar anaeróbio (mL.kg^-1^.min^-1^)	12.6±3
RER	1.12±0.8
Inclinação VE/VCO_2_	36±10
TD6 (passos)	85±47

BRA: bloqueador dos receptores da angiotensina; DAC: doença arterial coronariana; ECA: enzima conversora da angiotensina; NYHA: *New York Heart Association*; RER: razão de troca respiratória; VE/VCO_2:_ relação entre a ventilação e a produção de dióxido de carbono.

**Figura 1:**
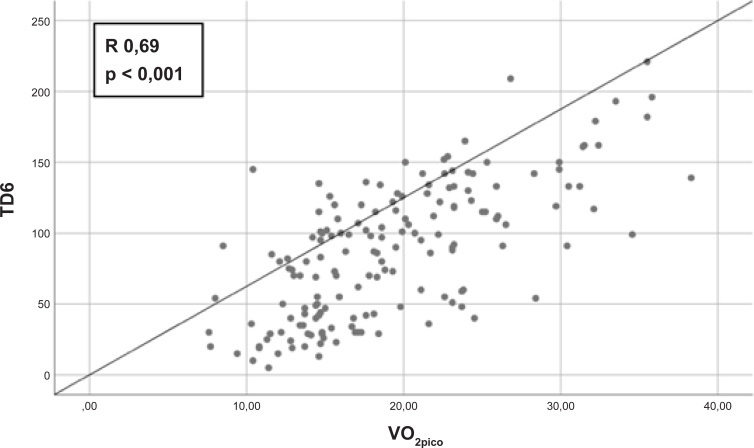
Associação entre o TD6 e o VO_2pico_.

**Figura 2:**
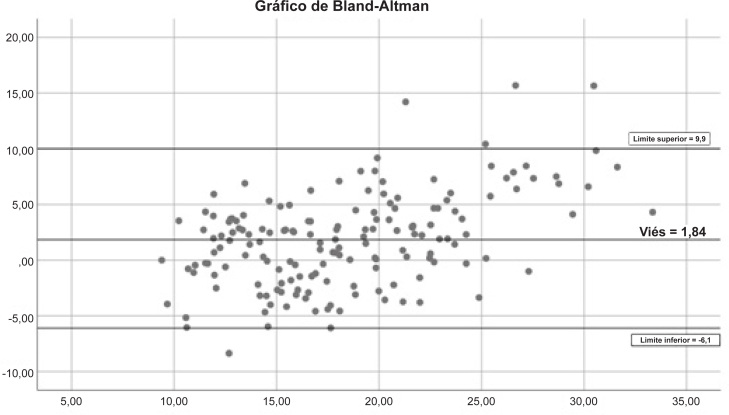
Gráfico de Bland-Altman para VO_2pico_ previsto vs. Determinado.

A associação entre o TD6 e o VO_2pico_ é apresentada na Figura 1; o índice de correlação r foi 0,69 (IC95% 0,60 – 0,78; p < 0,001), e o de R^2^ foi 0,47. A análise do gráfico de Bland-Altman é apresentada na Figura 2; a concordância estava distante do limite de referência superior ou inferior em apenas cinco pacientes.

Na análise multivariada, a idade, o sexo e os resultados do TD6 foram preditores independentes do VO_2pico_ (Tabela 2). As equações para a estimativa do VO_2pico_ com base no TD6 foram: VO_2pico_ = 19,6 + (0,075 x TD6) – (0,10 x idade) para homens e VO_2pico_ = 19,6 + (0,075 x TD6) – (0,10 x idade) – 2 para mulheres. O r ajustado do modelo final foi 0,72, e o R^2^ ajustado foi 0,53.

**Tabela 2:** Modelo final de regressão linear múltipla para predição do VO_2pico_ com base no TD6

Variável	Beta	Beta IC95%	p
TD6	0,075	(0,06) – (0,09)	< 0,001
Idade (anos)	-0,10	(-0,16) – (- 0,5)	< 0,001
Sexo feminino	-2,0	(-3,6) – (-0,33)	0,02
Constante	19,6	(15,2) – (24,1)	< 0,001

Ajustado para idade, fração de ejeção, doença arterial coronariana, insuficiência cardíaca e peso. IC: intervalo de confiança; TD6: teste do degrau de seis minutos.

**Figura 3:**
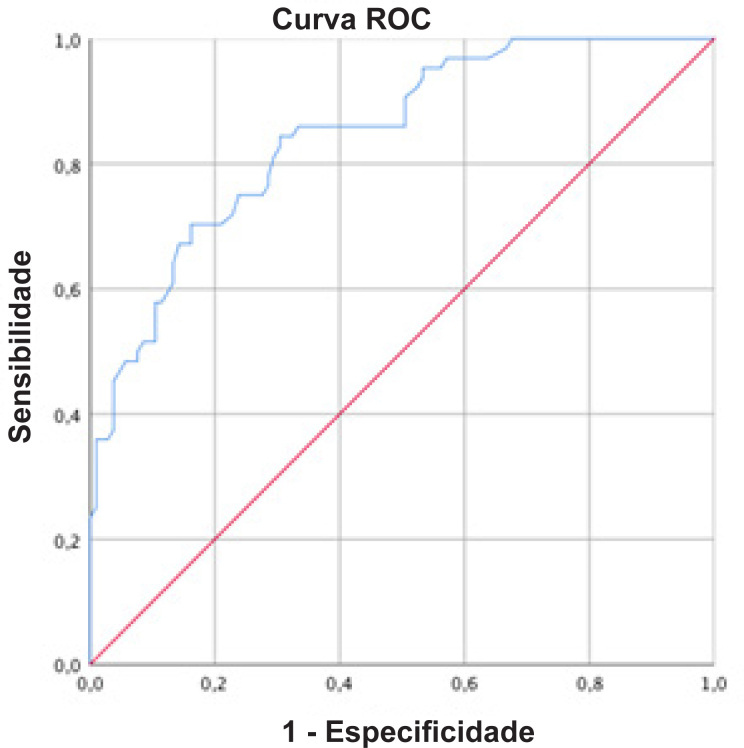
Curva ROC para que o TD6 preveja um VO_2pico_≥ 20 mL.kg^1-^.min^-1^. Área sob a curva 0.85 (IC95% 0,79 – 0,90) p < 0,001.

A curva ROC para o TD6 como preditor de VO_2pico_ ≥ 20 mL.kg^-1^.min^-1^ é apresentada na Figura 3. O ponto de corte mais acurado para que o TD6 preveja VO_2pico_ ≥ 20 mL.kg^-1^.min^-1^ foi > 105 passos (área sob a curva 0,85; IC95% 0,79-0,90; p <0,001).

## Discussão

A capacidade funcional é um dos parâmetros clínicos mais importantes para a avaliar a capacidade funcional.^1^ O comprometimento funcional está relacionado a pior prognóstico, independentemente do diagnóstico ou cenário clínico.^1^^,^^7^ A aptidão cardiorrespiratória (ACR) pode ser estimada por diversos métodos, embora o TECP seja o único método que permite determinação direta com base no VO_2pico_. Uma vez que o TECP requer equipamento específico e equipe médica bem treinada, uma medição indireta e acurada da capacidade funcional é muito desejável. É importante que sejam validados formulários alternativos mais simples para a avaliação de ACR, já que podem ser aplicados de forma mais ampla.

Em uma população de pacientes com DAC e IC, demonstramos que o TD6 apresentou boa correlação com o VO_2pico_, conforme medido pelo TCEP. Também conseguimos derivar uma equação para prever o VO_2pico_ com base nos resultados do TD6, bem como para determinar um ponto de corte para o número de passos necessários para identificar pacientes de baixo risco (valor mínimo do VO_2pico_ de 20 mL.kg^-1^.min^-1^).

### Teste do Degrau na Cardiologia

Os testes do degrau não são uma ferramenta nova na cardiologia. Na década de 1930, Master et al.,^8^ utilizaram um teste de escada de um degrau em um protocolo de 2 minutos para observar eletrocardiogramas de exercício. Esse foi o precursor dos atuais testes de esforço com ergômetros. O teste do degrau de Master era amplamente utilizado como teste provocativo para isquemia coronariana, mas não era rotineiramente utilizado como preditor de ACR/capacidade funcional e prognóstico. O principal objetivo do TD6 como teste submáximo é determinar a ACR e não diagnosticar isquemia coronariana. Assim como o TC6, o TD6 é seguro e pode ser realizado em esforço submáximo, embora com um gasto energético um pouco maior.

### Capacidade Funcional como Sinal Vital

A capacidade funcional pode ser considerada um sinal vital e deve ser avaliada em todas as consultas médicas.^9^^,^^10^ Pode ser prevista por testes de exercício regulares ou testes funcionais submáximos, como o TC6; no entanto, o TCEP é a única forma de avaliar e determinar diretamente a capacidade funcional. Com base em estudos clássicos em pacientes cardíacos, um VO_2pico_ acima de 20 mL.kg^-1^.min^-1^ é um marcador de bom prognóstico, independentemente de outros parâmetros. Por outro lado, aqueles com VO_2pico_ abaixo de 12 mL.kg^-1^.min^-1^ e IC podem ser considerados candidatos a transplante cardíaco.^5^^,^^6^

Como alternativa ao TCEP, o TC6 foi validado e é utilizado para avaliação prognóstica em diferentes doenças.^11^ É de fácil reprodução e pode ser relacionado ao desfecho, mas a necessidade de um espaço grande impede seu uso no consultório, por exemplo. Portanto, um teste que consiga estimar a capacidade funcional mesmo em espaço pequeno sem a necessidade de equipamentos sofisticados é de grande utilidade. É importante destacar que o TD6 foi previamente comparado ao TC6 em uma população sem doenças pulmonares ou cardíacas, apresentando boa correlação.^12^

O teste do degrau de seis minutos é uma forma simples de predizer capacidade funcional

O TD6 é um teste simples que não necessita de muito espaço. Pode ser realizado em um consultório médico ou por outros profissionais da saúde. O teste foi usado previamente em pacientes com doença pulmonar crônica, mas ainda não foi validado em pacientes cardíacos.

Em pacientes com doença pulmonar obstrutiva crônica, um ponto de corte de < 78 passos foi associado a pior prognóstico.^13^ Em uma população saudável com média de idade de 39 anos, a contagem média de passos foi 149±34.^14^

De acordo com nossos dados, o TD6 apresenta acurácia aceitável para prever o VO_2pico_ em uma amostra de pacientes com DAC/IC, e os profissionais de saúde podem querer usar esses resultados em sua prática clínica.

Observamos que o ponto de corte de > 105 passos está relacionado ao alcance de um VO_2pico_ acima de 20 mL.kg^-1^.min^-1^. Esse ponto de corte pode ser útil, por exemplo, quando o TCEP não estiver disponível. Além disso, se o paciente consegue subir mais de 105 degraus, o TCEP pode não ser necessário, pois estima-se um VO_2pico_ acima de 20 mL.kg^-1^.min^-1^.

As estimativas de capacidade funcional baseadas em atividades de rotina são imprecisas e não foram validadas diretamente por meio de dados do TCEP^15^ embora essa estratégia ainda seja utilizada quando estimativas imediatas são necessárias, mesmo para avaliação em série. Assim, o TD6 pode ser aplicado de forma fácil e rápida, porém com mais segurança no que se refere à determinação da capacidade funcional.

### Limitações

O presente estudo apresenta algumas limitações. Uma amostra maior e uma validação prospectiva dos resultados em outras populações devem ser consideradas. Nossa população consistiu em pacientes com DAC e/ou IC, os quais foram analisados em conjunto. Pode ser interessante analisar esses fenótipos separadamente. Para atenuar a influência do diagnóstico clínico na realização do teste, controlamos a análise multivariada para o diagnóstico de IC ou DAC e constatamos que o diagnóstico não influenciou o resultado. Também controlamos a análise para a fração de ejeção. Como a DAC é a causa mais prevalente de IC e a capacidade funcional é um fator prognóstico independente para ambos, a existência de um teste e um único ponto de corte que possa ser aplicado em um espectro mais amplo de doenças cardíacas pode ser de grande utilidade, já que o TD6 pode ser mais bem aplicado em triagem e acompanhamento.

A análise dos gráficos de Bland-Altman mostrou que a concordância foi considerada distante dos limites de controle superior ou inferior em apenas cinco pacientes. Desses, quatro apresentaram VO_2pico_ acima de 20 mL.kg^-1^.min^-1^, e o valor previsto no TD6 também foi maior de 20 mL.kg^-1^.min^-1^. Dessa forma, os quatro pacientes não seriam erroneamente classificados como de menor risco do que o esperado. Em um paciente, o VO_2pico_ previsto pelo TD6 foi maior do que o medido. Ao analisar esse caso, observamos que a razão de troca respiratória do TCEP apresentou compatibilidade de apenas 0,94 com um esforço submáximo, causada pela má adaptação à esteira e à máscara. O mesmo paciente subiu e desceu 91 degraus em 6 minutos. Pode-se entender que o TD6 é mais adequado como ferramenta de triagem e não como um substituto do TCEP. Portanto, embora sejam correlacionados, o TCEP ainda é necessário em casos em que a capacidade funcional precisa ser determinada com exatidão.

No momento, não temos dados de desempenho do TC6 para esses indivíduos, embora uma correlação entre os testes nesses pacientes possa ser útil. Estudos correlacionando o desempenho do TD6 em termos de desfechos clínicos devem fornecer mais informações sobre os melhores pontos de corte. Por fim, embora o TCEP VO_2pico_ seja o padrão-ouro para a avaliação funcional, é possível que o TD6 possa fornecer algumas implicações prognósticas, de acordo com os resultados.

## Conclusão

Foi derivada uma equação capaz de prever o VO_2pico_ com base nos resultados do TD6, e foi encontrada associação significativa entre o TD6 e o VO_2pico_. O ponto de corte do TD6, que prevê um VO_2pico_ ≥ 20 mL.kg^-1^.min^-1^, foi >105 passos.
