# Supplementary material for: O Teste do Degrau de Seis Minutos como Preditor de Capacidade Funcional de Acordo com o Consumo de Oxigênio de Pico em Pacientes Cardíacos
Source: Arq Bras Cardiol. 2021 May 6;116(5):889–95. [Article in Portuguese] doi: 10.36660/abc.20190624 (PMC8121484; doi:10.36660/abc.20190624)
Supplement: Translation

# Translation: The Six-Minute Step Test as a Predictor of Functional Capacity according to Peak VO_2_ in Cardiac Patients

**Authors:** Luiz Eduardo Fonteles Ritt, Eduardo Sahade Darzé, Gustavo Freitas Feitosa, Jessica Santana Porto, Gabriela Bastos, Renata Braga Linhares de Albuquerque, Cristiane Miura Feitosa, Thaissa Costa Claro, Eloisa Ferreira Prado, Queila Borges de Oliveira, Ricardo Stein

**Affiliations:** 1 SalvadorBA Brazil Hospital Cárdio Pulmonar, Salvador, BA - Brazil; 2 SalvadorBA Brazil Escola Bahiana de Medicina e Saúde Pública, Salvador, BA - Brazil; 3 Porto AlegreRS Brazil Hospital de Clínicas de Porto Alegre, , Porto Alegre, RS - Brazil; 4 Porto AlegreRS Brazil Universidade Federal do Rio Grande do Sul, Porto Alegre, RS - Brazil

**Keywords:** Heart Failure, Oxygen Consumption, Respiratoty Capacity, Tidal Volume, Exercise Test, Prognóstico, Mortalidade

## Abstract

**Background:**

Six-minute step test (6MST) is a simple way to evaluate functional capacity, although it has not been well studied in patients with coronary artery disease (CAD) or heart failure (HF).

**Objective:**

Analyze the association between the 6MST and peak oxygen uptake (VO_2peak_) and develop an equation for estimating VO_2peak_ based on the 6MST, as well as to determine a cutoff point for the 6MST that predicts a VO_2peak_ ≥20 mL.Kg^-1^.min^-1^

**Methods:**

In 171 patients who underwent the 6MST and a cardiopulmonary exercise test, correlation, regression, and ROC analysis were used and a p < 0.05 was admitted as significant.

**Results:**

mean age was 60±14 years and 74% were male. Mean left ventricle ejection fraction was 57±16%, 74% had CAD and 28% had HF. Mean VO_2peak_ was 19±6 mL.Kg^-1^.min^-1^ and mean 6MST performance was 87±45 steps. Association between 6MST and VO_2peak_ was r 0.69 (p <0.001). The model VO_2peak_ =19.6 + (0.075 x 6MST) – (0.10 x age) for men and VO_2peak_ =19.6 + (0.075 x 6MST) – (0.10 x age) – 2 for women could predict VO_2peak_ based on 6MST results (adjusted R 0.72; adjusted R^2^ 0.53). The most accurate cutoff point for 6MST to predict a VO_2peak_ ≥20 mL.Kg^-1^.min^-1^ was >105 steps (AUC 0.85; 95% CI 0.79 -0.90; p <0.001).

**Conclusion:**

An equation for predicting VO_2peak_ based on 6MST results was derived, and a significant association was found between 6MST and VO_2peak_. The cutoff point for 6MST, which predicts a VO_2peak_ ≥20 mL.Kg^-1^.min^-1^, was >105 steps. (Arq Bras Cardiol. 2021; 116(5):889-895)

## Introduction

In cardiovascular disease, functional capacity is directly related to prognosis.^1^ Functional performance, as determined by peak oxygen consumption (VO_2peak_) and measured with a cardiopulmonary exercise test (CPET), is the gold standard and is used to determine prognosis in heart failure (HF) and heart transplant selection, as well as to gauge therapeutic response.^2^^–^^4^ Patients with a VO_2peak_ below 15 mL.Kg^-1^.min^-1^ have a worse prognosis profile, and those with a VO_2peak_ above 20 mL.Kg^-1^.min^-1^ have a better prognosis profile, independent of HF etiology and ventricular function.^5^^,^^6^ Although widely used and validated, the CPET is not available in most centers, since the equipment is expensive and a specialized physician is required to administer the test and interpret its results.

One alternative to CPET is the six-minute walk test (6MWT), which is well validated and has a good correlation with CPET in cardiomyopathy patients.^7^ However, the 6MWT requires a long corridor (at least 30 meters), which could limit its use in normal practice.

The six-minute step test (6MST) is a simple test in which the patient climbs and descends a 2-step ladder for 6 minutes in free cadence, and the number of steps the patient takes is counted. It requires neither sophisticated equipment nor large spaces. Although studied in patients with chronic lung disease and in normal subjects,^8^^–^^11^ there are no data on 6MST performance in cardiac patients.

The objectives of this study were to: (1) analyze the association between 6MST and VO_2peak_, (2) develop an equation for estimating VO_2peak_ based on 6MST results, and (3) determine a cutoff point for lower risk category in the 6MST (VO_2peak_≥ 20 mL.Kg^-1^.min^-1^).

## Methods

In this cross-sectional study, we evaluated patients referred for cardiac rehabilitation between May 2014 and September 2017 who, as per clinical protocol, underwent symptom-limited CPET and 6MST as their baseline evaluation in the cardiac rehabilitation program of the Hospital Cárdio Pulmonar, Salvador, Brazil.

The inclusion criteria were patients older than 18 years who had been diagnosed with coronary artery disease (CAD) or heart failure (HF), characterized by previous acute myocardial infarction, coronary angioplasty/stent placed post-operatively to cardiac or vascular surgery, or patients with implantable devices such as pacemakers or cardiac defibrillators. These individuals were referred to the cardiac rehabilitation program and underwent an initial evaluation with a cardiologist and a physical therapist. CAD and/or HF diagnosis was established by medical history (acute myocardial infarction, stable CAD, myocardial revascularization or angioplasty, or symptoms of dyspnea or angina), electrocardiographic abnormalities (pathological Q waves) and echocardiographic abnormalities (ventricular dysfunction and segmental abnormalities).

The exclusion criteria were inability to perform the CPET or the 6MST. Patients with symptoms of angina or ischemia at an stage lower than the anaerobic threshold were also excluded as they were not submitted to the 6MST.

Clinical and demographic data were retrieved from the initial cardiologist evaluation on the day of the CPET, including the most recent (within the last 3 months) echocardiogram. The CPET and 6MST were applied separately, 2 to 7 days apart.

The 6MST was performed on a 20 cm high step covered with non-slip rubber. Patients were instructed to go up and down the step as fast as possible for 6 minutes without using their arms to support themselves; rest breaks were permitted.

A symptom-limited CPET was performed on a treadmill with a gas analyzer (Cortex, Leipzig, Germany) with breath-by-breath measurements. An individualized ramp protocol based on the functional class of each patient was used, having a targeted exercise phase duration between 8 and 12 minutes. The collected ventilatory data were tabulated and analyzed at 10-second intervals.

Ethical aspects: the study protocol was approved by the Celso Figueiroa Research Ethics Committee of the Hospital Santa Izabel (case 1.711.505). The study was conducted in accordance with national and international legislation for human research, including the Helsinki guidelines and resolution 466/12 of the Brazilian National Health Council. Informed consent was exempted since the study utilized only medical record data.

### Statistical Analysis

SPSS version 25.0 was used for all analyses. Continuous variables were presented as mean ± standard deviation (SD) for parametric distribution. The Shapiro-Wilk test and visual inspection of histograms were used to determine normality. Categorical variables were presented as proportion or percentage. The Pearson correlation was applied to determine associations between continuous variables and Bland-Altman plots to analyze their agreement. Univariate and multivariate linear regression analyses (after adequate assumptions were analyzed) were performed to determine the model’s prediction of VO_2peak_ based on the 6MST, which was controlled for age, ejection fraction, sex, presence of CAD or HF, and weight. ROC curve analysis was applied to determine the best cutoff points for predicting a VO_2peak_ ≥20 mL.Kg^-1^.min^-1^. A p-value <0.05 was considered significant for all analyses.

## Results

The total sample consisted of 171 patients. Table 1 shows their general demographic and clinical characteristics. Most patients were in NYHA class I or II (54% and 24%, respectively) with a mean VO_2peak_ of 19±6 mL.kg^-1^.min^-1^.

**Table 1:** General demographic and clinical characteristics of the population

Variable	Result
Male % (n)	74% (121)
Age (years)	60±14
CAD % (n)	74% (121)
Heart failure %(n)	28% (47)
Cardiac valvular disease %(n)	13% (22)
Diabetes %(n)	25% (44)
Hypertension %(n)	62% (102)
NYHA I, II, III%	53%/24%/10%
ACE inhibitor-ARB %(n)	65% (110)
Beta-blocker %(n)	77% (130)
Statins %(n)	75% (128)
Ejection fraction (%)	57±16
VO_2peak_ (mL.kg^-1^.min^-1^)	19±6
VO_2_ at anaerobic threshold (mL.kg^-1^.min^-1^)	12.6±3
RER	1.12±0.8
VE/VCO_2_ slope	36±10
6MST (steps)	85±47

CAD: coronary artery disease; NYHA: New York Heart Association; ACE: angiotensin receptor enzyme; ARB: angiotensin receptor blocker; RER: respiratory exchange ratio.

Figure 1 shows the association between 6MST and VO_2peak_; the r correlation index was 0.69 (95% CI 0.60 – 0.78; p < 0.001) and the R^2^ was 0.47. Figure 2 shows the Bland-Altman plot analysis, and in only 5 patients the agreement was away from the upper or the lower reference limit.

**Figure 1:** Association between 6MST and VO_2peak_.

**Figure 2:** Bland-Altman plot for predicted versus determined VO_2peak_.

In the multivariate analysis, age, sex and 6MST result were independent predictors of VO_2peak_ (Table 2). The equations for estimating VO_2peak_ based on 6MST were: VO_2peak_=19.6 + (0.075 x 6MST) – (0.10 x age) for men, and VO_2peak_=19.6 + (0.075 x 6MST) – (0.10 x age) – 2 for women. The final model’s adjusted r was 0.72 and the adjusted R^2^ was 0.53.

**Table 2:** Final multiple linear regression model for predicting VO_2peak_ based on the 6MST

Variable	Beta	Beta 95% CI	p
6MST	0.075	(0.06) – (0.09)	<0.001
Age (years)	-0.10	(-0.16) – (- 0.5)	<0.001
Female	- 2.0	(- 3.6) – (- 0.33)	0.02
Constant	19.6	(15.2) – (24.1)	<0.001

Adjusted for age, ejection fraction, coronary artery disease, heart failure, and weight. 6MST: six-minute step test; OR: odds ratio; CI: confidence interval.

Figure 3 shows the ROC curve for the 6MST as a predictor of VO_2peak_ ≥20 mL.kg^-1^.min^-1^. The most accurate cutoff point for 6MST prediction of VO_2peak_ ≥20 mL.kg^-1^.min^-1^ was >105 steps (AUC 0.85, 95% CI 0.79-0.90, p <0.001).

**Figure 3:** ROC curve for 6MST to predict a VO_2peak_ ≥ 20 mL.Kg^1-^.min^-1^ AUC 0.85 (95% CI 0.79 – 0.90) p < 0.001.

## Discussion

Functional capacity is one of the most important clinical parameters for measuring functional capacity.^1^ Functional impairment is related to worse prognosis, independently of the diagnosis or clinical scenario.^1^^,^^7^ Cardiorespiratory fitness (CRF) can be estimated by several methods, although the CPET is the only method that allows direct determination based on VO_2peak_. Since the CPET requires specific equipment and well-trained medical staff, an accurate indirect measurement of functional capacity is highly desirable. Simpler alternative forms of CRF evaluation are important to be validated as they may be more broadly applied.

In a population of CAD and HF patients, we demonstrated that the 6MST had a good correlation with VO_2peak_ as measured by CPET. We were also able to derive an equation to predict VO_2peak_ based on 6MST results, as well as to determine a cutoff point for the number of steps necessary to identify lower-risk patients (minimum VO_2peak_ of 20 mL.Kg^-1^.min^-1^).

### Step Test in Cardiology

Step tests are not a new tool in cardiology. Back in the 1930s, Master et al.^8^ used a 1-step stair test in a 2-minute protocol to observe exercise ECGs. This was the precursor of actual exercise stress tests using ergometers. The Master step test was widely used as a provocative test for coronary ischemia, but was not routinely used for predicting CRF/functional capacity and prognosis. The main purpose of the 6MST as a submaximal test is to determine CRF and not to diagnose coronary ischemia. As well as the 6MWT, the 6MST is safe and can be performed at submaximal effort, although with a slightly higher energy expenditure.

### Functional Capacity as a Vital Sign

Functional capacity can be considered a vital sign and should be assessed in each clinical visit.^9^^,^^10^ It can be predicted with a regular exercise test or submaximal functional tests such as the 6MWT, but the CPET is the only way to directly assess and determine functional capacity. Based on classic studies of cardiac patients, a VO_2peak_ above 20 mL.kg^-1^.min^-1^ is a marker of good prognosis regardless of other parameters, whereas those with a VO_2peak_ below 12 mL.kg^-1^.min^-1^ and HF may even be considered candidates for heart transplant.^5^^,^^6^

As an alternative to CPET, the 6MWT has been validated and is used for prognostic evaluation in different diseases.^11^ It is easy to replicate and can be related to outcome, but the requirement of a large space prevents its use in office settings. Therefore, a test that can estimate functional capacity in the office without the need for sophisticated equipment is of value.

It is important to point out that the 6MST was previously compared to the 6MWT in a population free of cardiac or pulmonary diseases and showed good correlation.^12^

### The 6-minute Step Test is a Simple Way to Predict Functional Capacity

The 6MST is a simple test that does not require much space. It can be applied in a medical office or by other health professionals. It has been previously used in patients with chronic pulmonary disease, but it has not yet been validated in cardiac patients.

In patients with chronic obstructive pulmonary disease, a cutoff point of <78 steps was associated with worse prognosis.^13^ In a healthy population with a mean age of 39 years, the mean step count was 149 ± 34.^14^

According to our data, the 6MST has acceptable accuracy for predicting VO_2peak_ in a sample of CAD/HF patients, and clinicians may want to use these results in their clinical practice.

We found that the cutoff point of >105 steps is related to achieving a VO_2peak_ above 20 mL.kg^-1^.min^-1^. This cutoff may be useful, for example, when CPET is not available. Furthermore, if a patient can climb more than 105 steps, a CPET may not be necessary, since a VO_2peak_ above 20 mL.kg^-1^.min^-1^ is estimated.

Functional capacity estimates based on daily life activities are inaccurate and have not been directly validated through CPET data,^15^ although this strategy is still used when prompt estimates are necessary, even for serial evaluation. Thus, the 6MST can be easily and quickly applied, but with more confidence regarding the determination of functional capacity.

### Limitations

This study has some limitations. A larger sample size and prospective validation of these results in other populations should be considered. Our population consisted of CAD and/or HF patients who were analyzed together, and one may want to analyze these phenotypes separately. To mitigate the influence of the clinical diagnosis on the performance of the test, we controlled the multivariate analysis for the diagnosis of HF or CAD and found that the diagnosis did not influence the result. Also, we controlled the analysis for ejection fraction. As CAD is the most prevalent cause of HF and functional capacity is an independent prognostic factor for both, having a test and a single cutoff point that can be applied to a broader spectrum of heart disease may be of value as the 6MST may be better applied to triage and follow-up. The Bland-Altman plot analysis showed that in only 5 patients the agreement was considered away from the upper or lower reference limits. Of these, 4 patients had VO_2peak_ above 20 mL.kg^-1^.min^-1^ and the predicted value from the 6MST was also greater than 20 mL.kg^-1^.min^-1^. Thus, these 4 patients would not be misclassified as having a lower risk than expected. In 1 patient, the VO_2peak_ predicted by the 6MST was higher than the measured one. By analyzing this case, we note that the CPET respiratory exchange ratio was just 0.94 compatible with a submaximal effort, which was due to poor adaptation to the treadmill and mask. This same patient climbed up and down 91 steps in 6 minutes. One may understand that the 6MST is more suitable as a triage tool than as a substitute for the CPET. Therefore, although they are correlated, in cases in which functional capacity needs to be exactly determined, the CPET is still needed. At the present moment, we have no 6MWT performance data for these individuals, although a correlation between the tests in these patients could be of value. Studies correlating the performance of the 6MST in terms of clinical outcomes should provide more information about the best cutoff points. Finally, although CPET VO_2peak_ is the gold standard for functional evaluation, it is possible that the 6MST could provide some prognostic implications according to the results.

## Conclusion

An equation capable of predicting VO_2peak_ based on 6MST results was derived, and a significant association was found between 6MST and VO_2peak_. The cutoff point for the 6MST, which predicts a VO_2peak_ ≥ 20 mL.Kg^-1^.min^-1^, was >105 steps.
